# Advanced Imaging Techniques for Newly Diagnosed and Recurrent Gliomas

**DOI:** 10.3389/fnins.2022.787755

**Published:** 2022-02-23

**Authors:** Luis R. Carrete, Jacob S. Young, Soonmee Cha

**Affiliations:** ^1^University of California San Francisco School of Medicine, San Francisco, CA, United States; ^2^Department of Neurological Surgery, University of California, San Francisco, San Francisco, CA, United States; ^3^Department of Radiology, University of California, San Francisco, San Francisco, CA, United States

**Keywords:** glioma, imaging, recurrence, progression, pseudoprogression, radiomics, PET scanning

## Abstract

Management of gliomas following initial diagnosis requires thoughtful presurgical planning followed by regular imaging to monitor treatment response and survey for new tumor growth. Traditional MR imaging modalities such as T1 post-contrast and T2-weighted sequences have long been a staple of tumor diagnosis, surgical planning, and post-treatment surveillance. While these sequences remain integral in the management of gliomas, advances in imaging techniques have allowed for a more detailed characterization of tumor characteristics. Advanced MR sequences such as perfusion, diffusion, and susceptibility weighted imaging, as well as PET scans have emerged as valuable tools to inform clinical decision making and provide a non-invasive way to help distinguish between tumor recurrence and pseudoprogression. Furthermore, these advances in imaging have extended to the operating room and assist in making surgical resections safer. Nevertheless, surgery, chemotherapy, and radiation treatment continue to make the interpretation of MR changes difficult for glioma patients. As analytics and machine learning techniques improve, radiomics offers the potential to be more quantitative and personalized in the interpretation of imaging data for gliomas. In this review, we describe the role of these newer imaging modalities during the different stages of management for patients with gliomas, focusing on the pre-operative, post-operative, and surveillance periods. Finally, we discuss radiomics as a means of promoting personalized patient care in the future.

## Introduction

Gliomas are the most common primary brain tumor with varying prognosis depending on their grade and genomic profile (Louis et al., 2021). Patients often present with seizures and/or focal neurological deficit and undergo an imaging study, often an MRI scan, that reveals the neoplastic lesion. The standard of care for these patients begins with maximal safe resection followed by chemotherapy and radiation (Stupp et al., 2005; Molinaro et al., 2020). In the case of high grade glioma (HGG), there is substantial evidence to suggest that maximal extent of resection (EOR) of the contrast-enhancing (CE) regions of tumor on post-contrast T1 imaging, and in some cases resection of non-contrast enhancing (NCE) disease that extends beyond the CE tumor (i.e., a supratotal resection), improves survival (Li et al., 2016; Esquenazi et al., 2017; Molinaro et al., 2020).

In order to achieve a maximal safe resection of these infiltrative tumors, particularly when the goal is a supratotal resection, advanced imaging modalities, such as perfusion imaging, diffusion imaging, spectroscopy, and positron emission topography (PET) imaging, have become increasingly useful surgical adjuncts, especially when used in combination with intraoperative brain mapping. Nevertheless, despite aggressive treatments, these tumors almost always recur, and early detection of tumor recurrence remains critical for optimal patient management and evaluation of treatment options, including feasibility of repeat surgical resection. In the months following tumor resection and radiation therapy, the appearance of treatment effect or pseudoprogression, which is defined as the radiographic appearance of tumor growth that spontaneously resolves without additional anti-tumor therapy and is actually reflective of treatment response, can closely resemble the appearance of recurrent, progressive tumor on imaging. Therefore, it is critical to distinguish between these phenomena to best manage patients (Stupp et al., 2005, 2009, 2014). Additionally, distinguishing true progression from pseudoprogression is critical for proper patient enrollment in clinical trials at the time of recurrence. This distinction represents another key area where multimodal imaging studies can improve clinical decision-making (Chaskis et al., 2009). In this review, we describe the role for various imaging modalities for patients with primary brain tumors during the pre-operative and surveillance stages of treatment and highlight the emerging field of radiomics for gliomas.

## Preoperative Imaging and Surgical Planning

After the initial diagnosis of lesion concerning for an intra-axial brain tumor is made, most institutions employ a standardized protocol for lesion characterization and pre-operative planning. 3-dimensional (3D) T1 pre- and post-contrast-enhanced, T2 contrast-enhanced, fluid-attenuated inversion recovery (FLAIR), and diffusion-weighted imaging (DWI) at a magnetic field strength of a minimum of 1.5 tesla (T) are commonly a part of these protocols (Ellingson et al., 2015). In the following section, we describe these common sequences and discuss how they can be used to clarify the diagnosis, determine the extent of tumor invasion, and maximize safety during tumor resection. For an overview of the various imaging techniques covered in this review, see Table 1.

**TABLE 1:** Imaging techniques for glioma imaging, utility, and limitations.

MRI technique	Clinical utility and findings
**Preoperative Techniques for Tumor Characterization**	
T1	Anatomic MRI- evaluates tissue architecture
Pre-contrast	Hyperintensity from fat, blood products, mineralization
Post-contrast	Demonstrates non-specific BBB breakdown
T2/FLAIR	Anatomic MRI- evaluates tissue architecture
	Hyperintensity in peritumoral edema, non-enhancing tumor, gliosis, white matter injury
DWI	Evaluates Brownian motion/diffusion of water molecules, can be presented as an ADC map
	Reduced diffusion (high signal intensity) in areas of increased cellularity due to tumor and in cytotoxic edema or postoperative injury
SWI	Sensitive to magnetic susceptibility of tissues
	Hypointense appearance from blood products, hyperintense appearance from calcification
MRS	Evaluate tumor biochemical/metabolic profile
	HGGs show higher Cho/NAA and Cho/Cr ratios than LGGs
**Perfusion imaging**	
DSC	Main metric is cerebral blood volume
	High blood volume suggestive of higher tumor grade or tumor recurrence
DCE	Main metric is k^trans^, a measure of permeability
	High permeability suggests higher tumor grade
ASL	Main metric is cerebral blood flow
	High blood flow suggestive of higher-grade tumor. Does not require exogenous contrast.
PET	Investigates tumor rates of proliferation and metabolism using molecular tracers
FDG PET	Compares rates of tumor uptake of glucose metabolism relative to surrounding tissue; higher rates of glucose metabolism seen in higher tumor grades
AA PET	Compares rates of amino acid transport in tumors relative to surrounding brain tissue; higher rates of amino acid tracer metabolism indicative of higher tumor grade.
**Imaging techniques for preoperative and intraoperative**	
DTI	Examines the direction of diffusivity of water molecules along white matter tracts.
	Tractography demonstrates location of white matter tracts relative to infiltrative tumor to inform pre- and intra-operative planning
fMRI	Evaluate brain activation based on specific tasks based on regional changes in blood oxygenation levels
	Used for functional mapping of specific brain regions to help preserve areas critical to perform certain tasks by is limited by poor sensitivity and specificity and overall poor correlation with intraoperative direct electrical stimulation mapping
MEG	Detects magnetic fields generated by electrical currents from neuronal action potentials
	Registered with 3D MRI sequence to visualize functional neuronal activity
nTMS	Utilizes transcranial magnetic fields to non-invasively stimulate/inhibit brain cortex
	Transcranial magnetic fields applied through non-invasive image-guided method to generate functional maps to differentiate eloquent from non-eloquent cortex.

*HGG, high-grade glioma; LGG, low-grade glioma; MRI, magnetic resonance imaging; FLAIR, fluid-attenuated inversion recovery; BBB, blood-brain barrier; DWI, diffusion-weighted imaging; SWI, susceptibility-weighted imaging; DSC, dynamic susceptibility contrast; DCE, dynamic contrast enhanced; ASL, arterial spin labeling; MRS, MR spectroscopy; DTI, diffusion tensor imaging; fMRI, functional MRI; MEG, magnetoencephalography; nTMS, navigated transcranial magnetic stimulation; PET, positron emission tomography; FDG PET, 2-^18^F-fluoro-2-deoxy-D-glucose; AA PET, amino acid PET.*

### T1 Pre- and Post-gadolinium and T2/T2 Fluid-Attenuated Inversion Recovery Sequences

**FIGURE 1:**
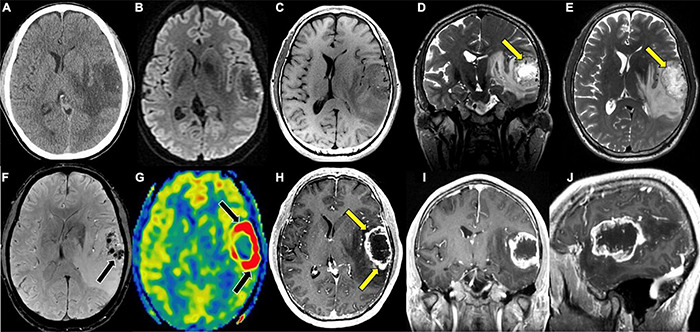
Glioblastoma, IDH-wildtype. **(A)** Axial CT without contrast: Ill-defined hypodensity lesion centered in the left superior temporal gyrus; **(B)** axial DWI: No associated reduced diffusion; **(C)** axial T1 pre-contrast: hypointense mass; **(D)** coronal T2: heterogeneous mass with hypointense rim with prominent central necrosis (yellow arrow); **(E)** axial T2: heterogeneous mass with hypointense rim with prominent central necrosis (yellow arrow); **(F)** axial SWI: prominent blood products within the mass (black arrow); **(G)** axial ASL perfusion: marked hyperperfusion (black arrows) within the rim enhancing component of the mass; **(H)** axial T1 post-contrast: heterogeneous mass with thick rim enhancement (yellow arrows) and prominent central necrosis; **(I)** coronal T1 post-contrast: heterogeneous mass with thick rim enhancement and prominent central necrosis; **(J)** sagittal T1 post-contrast: heterogeneous mass with thick rim enhancement and prominent central necrosis. CT, Computed tomography; DWI, Diffusion-weighted imaging; SWI, Susceptibility-weighted imaging; ASL, Arterial spin labeling; MRS, Magnetic resonance spectroscopy.

T1-weighted pre- and post-contrast images as well as the T2 weighted images, particularly the fluid-attenuated inversion recovery (T2 FLAIR) sequence, are often the most critical for tumor visualization and are most frequently utilized intra-operatively with neuronavigation to assist with tumor resection. Aberrant vascular proliferation and tumor necrosis caused by high grade gliomas results in disruption of the blood-brain barrier (BBB) and vascular leakage of intravenously administered gadolinium contrast agent (Upadhyay and Waldman, 2011; Ellingson et al., 2017a; Hu et al., 2020). The contrast agent extravasation leads to T1 shortening and hyperintensity (i.e., “contrast-enhancement”) on T1-weighted imaging (Figure 1; Hervey-Jumper and Berger, 2016; Hu et al., 2020).

In the case of glioblastoma (GBM), it is well-known that malignant cells invade past areas of contrast enhancement on T1 imaging (Berman et al., 2007). As mentioned above, a recent multicenter cohort study investigating maximal resection of CE and NCE tumors demonstrated that in addition to the survival benefit conferred by maximal resection of the CE tumor, additional resection of NCE tumor leads to improved overall survival (OS) regardless of isocitrate dehydrogenase (IDH) and O6-methylguanine-DNA methyltransferase (MGMT) methylation status in younger patients (Molinaro et al., 2020). This underscores the need for imaging modalities for HGG delineating tumor infiltration past areas of high contrast enhancement.

**FIGURE 2:**
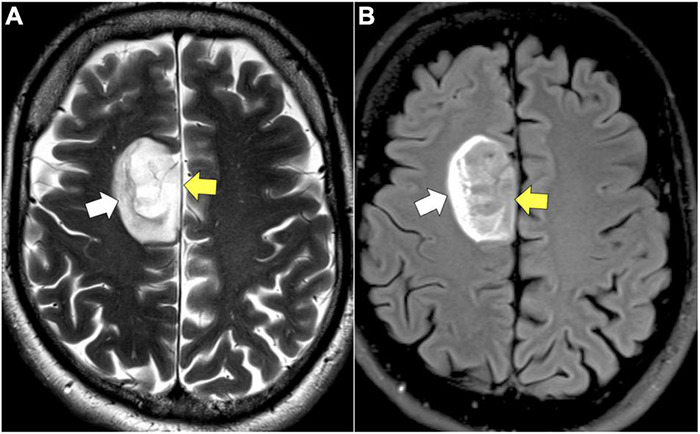
T2/FLAIR mismatch. **(A)** Axial T2: homogenously hyperintense mass (yellow arrow). **(B)** Axial FLAIR: hypointense mass (yellow arrow) relative to T2 image with exception of a hyperintense peripheral rim (white arrow).

T2/FLAIR sequences are better suited for visualizing low-grade glioma (LGG) as well as areas of edema and tumor growth extending past CE areas on T1 typical of HGG. Low-grade tumors less frequently enhance on T1 post-contrast images given their lower rates of proliferation and intact BBB, making T2/FLAIR an important sequence in the evaluation of LGG (Figure 1; Sage and Wilson, 1994; Scott et al., 2002; Harpold et al., 2007; Gupta and Dwivedi, 2016). When LGGs do contrast enhance, the pattern is often patchy or wispy, which can indicate areas of malignant transformation (Whitfield et al., 2014; Zhang et al., 2021). Unlike, T1 sequences, T2/FLAIR functions by demonstrating hyperintensity in areas of prolonged transverse relaxation time due to increased water content (Upadhyay and Waldman, 2011; Ellingson et al., 2017a; Hu et al., 2020; Li J. et al., 2020). This property is useful in the visualization of peritumoral edema, an area containing infiltrating tumor cells and increased extracellular water due to plasma fluid leakage from aberrant tumor capillaries that surrounds the CE tumor core in HGG (Saadoun et al., 2002; Warth et al., 2007; Barajas et al., 2012, 2013). T2/FLAIR thus plays a valuable role in the planning of HGG resection given it reveals NCE areas of infiltrative disease (Barajas et al., 2012; Henker et al., 2019; Hu et al., 2020; Verburg and de Witt Hamer, 2021). T2 and FLAIR sequences can also be used to predict clinically relevant molecular features of glioma, as is the case with T2/FLAIR mismatch sign. T2/FLAIR mismatch sign is characterized by a the presence of areas of hyperintensity on T2-weighted image paired with a relatively hypointense signal on FLAIR imaging with the exception of a hyperintense peripheral rim (Figure 2; Deguchi et al., 2020). This finding is strongly indicative of IDH-mutant, 1p/19q non-codeleted astroyctomas with a positive predictive value (PPV) ranging from 83 to 100% (Patel S. H. et al., 2017; Broen et al., 2018; Deguchi et al., 2020).

While T2 FLAIR and T1 post-contrast images- provide crucial information in the preoperative and intraoperative period, there are limitations to the information provided by these modalities. Despite the utility of T1-post contrast imaging for detecting HGG based on contrast extravasation, tumors such as GBM occasionally show no or minimal enhancement on T1 post-contrast imaging (Figure 1). Similarly T2 and FLAIR sequences are limited in their ability to distinguish between LGG and HGG (Scott et al., 2002; Maia et al., 2004). These limitations are addressed to a degree through the use of additional sequences and imaging modalities that serve to complement anatomic MR sequences in the assessment of gliomas to further characterize tumor grade.

### Diffusion-Weighted Imaging

**FIGURE 3:**
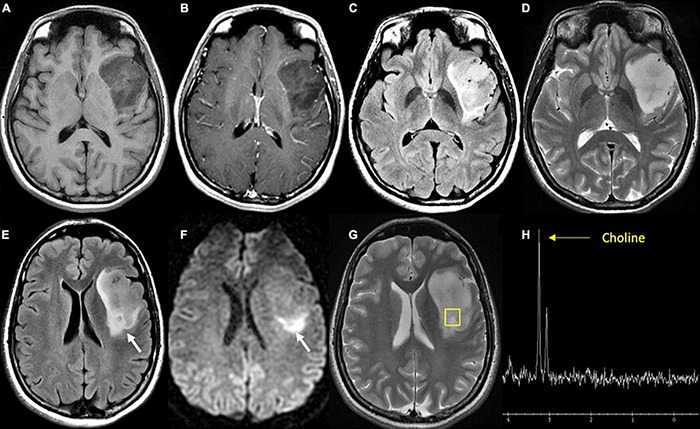
Diffuse astrocytoma, IDH-wildtype. **(A)** Axial T1 pre-contrast: expansile hypointense left insular mass; **(B)** Axial T1 post-contrast: No associated enhancement; **(C)** axial FLAIR: heterogeneous mixed hyper- and hypointense signal intensity within the mass; **(D)** axial T2: homogeneous hyperintense mass; **(E)** axial FLAIR: hyperintense region of the tumor (white arrow) in the posterior aspect; **(F)** axial DWI: associated reduced diffusion in the posterior tumor (white arrow); **(G)** axial T2: localizer for single voxel MRS targeted to the posterior tumor; **(H)** proton MRS single voxel: pathologic increase in choline metabolite at 3.2 ppm (yellow arrow) and absent NAA metabolite (arrowhead) at 2 ppm consistent with proliferating process. Biopsy targeted to this region showed cellular astrocytoma. FLAIR, Fluid-attenuated inversion recovery.

Diffusion-weighted imaging (DWI) is an MR sequence that measures random (Brownian) movement of water molecules and calculates diffusion metrics such as apparent diffusion coefficient (ADC). In the context of glioma imaging, restricted diffusion (i.e., low ADC signal) indicates hypercellularity due to high tumor proliferation and restriction of water diffusion compared to tissues with lower cellular density. These areas of hypercellularity causing restricted water diffusion appear as bright signal on DWI (Figure 3; Schmainda, 2012). Several studies demonstrate an inverse relationship between ADC and cellular density with this inverse relationship also existing for ADC in relation to tumor proliferation (Ellingson et al., 2010; Schmainda, 2012). Notably, intratumoral heterogeneity and areas of necrosis can limit the utility of ADC values in certain regions of the tumor (Lam et al., 2002). Still, this property of detecting hypercellularity can detect early stages of malignant transformation that may not yet show contrast enhancement on T1 (Baehring et al., 2007; Schmainda, 2012).

A more recently developed technique of diffusion imaging known as diffusion kurtosis imaging (DKI) functions by quantifying the non-Gaussian nature of water molecules to develop several metrics that allow for a significantly better characterization of intratumoral heterogeneity relative to DWI. Recent studies propose that DKI is capable of detailing certain differences between tumor grades in gliomas, including differences between WHO grades II and III, and differences between grades III and IV (Raja et al., 2016). This emerging technology is not commonly used in clinical practice, but may play a role as a non-invasive means of evaluating a tumor’s molecular characteristics (Raja et al., 2016).

### Susceptibility-Weighted Imaging

Susceptibility-weighted imaging (SWI) is an MR sequence that shows differences in the local magnetic field susceptibility among neighboring tissues, allowing for visualization of substances such as deoxyhemoglobin in venous blood, iron deposition in the brain, or calcium, providing important diagnostic information (Löbel et al., 2010). SWI images are generated by processing data that is acquired from methodology that includes a combination of high-resolution (3.0 T), a long echo time (TE), full-flow compensation, and a 3D gradient-echo. The acquired MR data is processed to detect susceptibility differences in substances within tissues such as those that are ferromagnetic (iron), paramagnetic (deoxyhemoglobin, clots), and diamagnetic (calcium) (Haacke et al., 2004). Magnetic fields applied to diamagnetic calcium appear bright while paramagnetic blood products appear dark on imaging (Figure 1). Based on these properties, SWI is the preferred modality for visualizing microhemorrhages, which becomes particularly valuable when trying to distinguish areas of necrosis or hemorrhage after radiation treatment, which will be discussed in more detail later in the review (Li et al., 2010; Löbel et al., 2010; Mohammed et al., 2013). The ability of SWI to identify calcium on imaging can also aid in predicting tumor histology and/or grade prior to pathological confirmation, particularly if a CT scan has not been obtained. Appearing as areas of low signal intensity, intratumoral calcification is most common in oligodendrogliomas, and can also be seen in gangliogliomas, pilocytic astrocytomas, and ependymomas (Emblem et al., 2008; Mohammed et al., 2013; Hsu et al., 2016). Additionally, there are studies that demonstrate that SWI can help differentiate brain abscess and necrotic GBM in cases where it is difficult to make a distinction on T1 post-contrast imaging (Toh et al., 2012).

In terms of limitations of this technique, multiple studies report long acquisition time of SWI as a limitation of this modality given that prolonged imaging can cause patient discomfort and is susceptible to motion artifact and image distortion (Sehgal et al., 2006; Tong et al., 2008; Fujima et al., 2010). Furthermore, while SWI is adept at imaging blood products and can depict blood vessels in both low-grade and high-grade glioma, the presence of calcium or hemorrhage within the tumor can serve as a susceptibility artifact that leads to underestimation of tumor perfusion; therefore, SWI is not as commonly used for visualizing microvessel density and tumor perfusion as other MR techniques (Willats and Calamante, 2013).

### MR Perfusion Imaging

**FIGURE 4:**
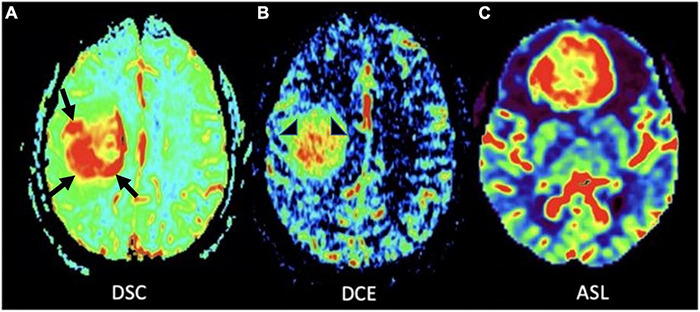
MR perfusion sequences. **(A)** Axial DSC perfusion: Marked hyperperfusion within the lateral and posterior aspects of the mass (black arrows); **(B)** axial DCE perfusion: Marked capillary leakiness within the central aspect of the mass (arrowheads); **(C)** axial ASL perfusion: Marked hyperperfusion of a neoplasm in the frontal lobe. DSC, Dynamic susceptibility-weighted contrast-enhanced; DCE, Dynamic contrast enhanced; ASL, Arterial spin labeling.

Dynamic susceptibility contrast (DSC), dynamic contrast enhanced (DCE), and arterial spin labeling (ASL) are the most commonly used MR perfusion techniques in clinical practice. DSC works by measuring transient decrease in brain signal intensity on T2*-weighted sequences after gadolinium contrast agent administration to generate a signal intensity time curve that is used to compute relative cerebral blood volume (rCBV) for each voxel (Hu et al., 2012). rCBV signal correlates directly with microvessel volume and is seen as a marker of angiogenesis (Figure 4), serving to distinguish HGG from LGGs and non-neoplastic etiologies such as post-treatment effect with greater rCBV signal in higher grade gliomas (Danchaivijitr et al., 2008; Xiao et al., 2015). There is evidence to suggest that this modality is predictive of malignant transformation of LGGs (Maia et al., 2004) and overall survival, and can be particularly helpful in distinguishing tumor recurrence from post-treatment effect such as radiation necrosis, and pseudoprogression- defined as a transient increase in post-contrast enhancement within the treated tumor lesion that presents during the first 6 months after treatment, followed by spontaneous radiographic improvement or resolution without any changes to the treatment regimen (Law et al., 2006, 2008; Chaskis et al., 2009; Schmainda, 2012; van West et al., 2017).

The absolute quantification of cerebral blood flow, cerebral blood volume, and mean transit time using DSC is dependent on what is known as the arterial input function (AIF). The AIF describes contrast agent input to the brain through the measurement of contrast agent concentration as it flows through brain-feeding arteries. In clinical practice AIF is typically obtained by manually selecting a region of interest around a feeding artery (i.e., the internal carotid artery or middle cerebral artery), a process that is subjective, user-dependent, and can lead to variability between scans, affecting repeatability of DSC studies (Bleeker et al., 2011; Jafari-Khouzani et al., 2015).

**FIGURE 5:**
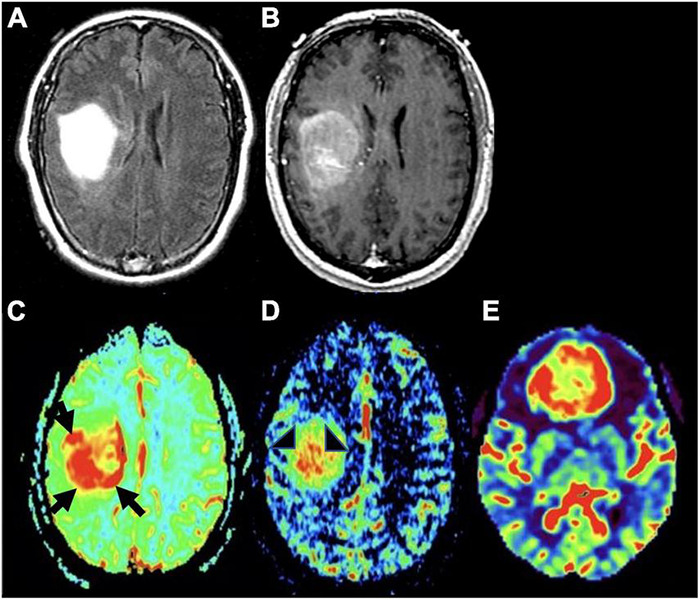
Molecular glioblastoma. **(A)** Axial FLAIR: homogeneously hyperintense mass; **(B)** axial T1 post-contrast: mild enhancement within the mass without distinct area of necrosis; **(C)** axial DSC perfusion: marked hyperperfusion within the lateral and posterior aspects of the mass (black arrows); **(D)** axial DCE perfusion: marked capillary leakiness within the central aspect of the mass (arrowheads); **(E)** axial ASL perfusion: marked increase in cerebral blood flow and hyperperfusion of tumor in the frontal lobe (different tumor than the one depicted in panels **A–D**).

DCE works by measuring dynamic signal changes on T1 weighted imaging after administration of intravenous contrast. In the context of glioma imaging, DCE is used to measure rates of capillary permeability caused by disordered tumor vasculature through the use of a volume transfer constant- k^trans^. This metric can be informative in the process of tumor grading given that HGGs have greater vascular permeability relative to LGGs (Roberts et al., 2000; Patankar et al., 2005). Given that DCE is primarily used to provide insight into tumor vascular permeability (Figures 4, 5), the utility of DCE is limited for the imaging of gliomas that do not exhibit BBB disruption or vascular leakage (Patankar et al., 2005; Essig et al., 2013).

**FIGURE 6:**
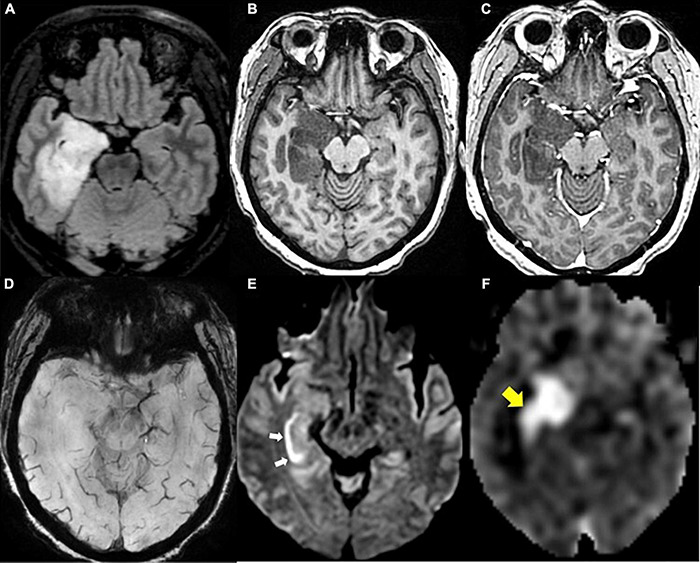
Diffuse glioma, IDH-mutant. **(A)** Axial FLAIR: Expansile hyperintense mass in the right medial temporal lobe; **(B)** axial T1 pre-contrast: hypointense mass; **(C)** axial T1 post-contrast: no associated enhancement within the mass; **(D)** axial SWI: no blood products or calcium within the mass; **(E)** axial DWI: linear reduced diffusion (white arrows) in the right hippocampus due to recent seizure activity; **(F)** axial ASL perfusion: Marked hyperperfusion within the right hippocampus and medial temporal lobe due to recent seizure activity (yellow arrow).

In contrast to DSC and DCE, ASL does not require intravenous injection of exogenous contrast (Figures 1, 4, 6). This technique quantitatively measures cerebral blood flow by inverting the magnetization of water protons in blood with a train of radiofrequency pulses in the carotid or vertebral arteries before blood enters the brain. Images are then collected and subsequently subtracted from a set of control static images, allowing for the quantification of cerebral blood flow (Zhang et al., 1995; Villanueva-Meyer et al., 2017; Hernandez-Garcia et al., 2019; Williams et al., 2019). Based on these properties, ASL can be used to differentiate HGG from LGG based on elevated perfusion (Kim et al., 2008; Cebeci et al., 2014; Villanueva-Meyer et al., 2017).

On the other hand, ASL is limited by a low signal-to-noise ratio that results from the fact that labeled molecules in blood make up only 0.5–1.5% of the full static tissue signal, which can be improved by increasing total scan time. Additionally, absolute CBF quantification using ASL can be highly variable between patients due to differences in physiologic factors such as cardiovascular disease, age, sex, and hematocrit (Henriksen et al., 2013). Furthermore, a lack of standardization in postprocessing algorithms can result in variability in CBF measurements (Delgado et al., 2018). In order to develop a protocol for the widespread clinical use of ASL in the distinction between LGGs and HGGs, further standardization of absolute CBF quantification methods is warranted.

Of note, there are additional techniques that have emerged in more recent years that also can provide valuable information about glioma perfusion that are currently being explored as methods of improving the diagnostic accuracy of imaging. One such example is that of vessel architecture imaging (VAI) MRI, a technique that serves to describe the structural heterogeneity of microvasculature in the brain. VAI is used to examine differences in tumoral vasculature based on glioma type that helps in the distinction of LGG from HGG (Zhang et al., 2019).

Another technique that has been adopted in the imaging of gliomas is intravoxel incoherent motion (IVIM)- a technique that captures data on perfusion and diffusion of water molecules in brain tissue and associated capillary networks (Le Bihan et al., 1986; Shen et al., 2016; Togao et al., 2016; Zou et al., 2018). This sequence does not require the injection of exogenous contrast, and is utilized in certain studies to attempt to characterize glioma grade and IDH1 mutational status (Wang et al., 2019). Although these modalities are not used commonly in clinical practice, they may prove useful as adjuncts to the aforementioned MR perfusion techniques that are more widely used (DSC, DCE, and ASL).

### MR Spectroscopy

MR spectroscopy (MRS) helps characterize the biochemical composition of regions of interest in the brain. This includes steady state concentrations of certain metabolites, metabolic reaction rates, and transport between cellular compartments. MRS provides insight into differences in biochemical composition between normal brain tissue and tumor. By tracking the presence of certain metabolites, such as choline (Cho), *N*-acetylaspartate (NAA), creatinine (Cr), lactate (Lac), and myo-inositol (MI), MRS provides insight into cell membrane turnover (Cho), neuronal viability (NAA), normal cellular metabolism (Cr), tissue hypoxia (Lac), and astrocytic integrity (MI) (Herholz et al., 1992; Castillo et al., 2000; Gupta et al., 2000; Barker, 2001; Villanueva-Meyer et al., 2017). In the context of preoperative glioma assessment, Cho/NAA and Cho/Cr metabolite peaks exhibit a positive correlation with increasing tumor grade (Figure 3), and can be used to distinguish vasogenic edema from infiltrative edema (Fountas et al., 2004; Hourani et al., 2008; Villanueva-Meyer et al., 2017; Hu et al., 2020). Furthermore, emerging MRS technology allows for the detection of 2-hydroxyglutarate (2-HG), an oncometabolite that is produced by IDH mutant tumor cells serving as a non-invasive assessment of lesions where the diagnosis of LGG is uncertain (Choi et al., 2012). Histologically validated studies show that 2-HG detection through MRS can be used to distinguish IDH mutant from IDH wildtype glioma, albeit with variable sensitivity and specificity (Choi et al., 2016; Tietze et al., 2018). Limitations to this modality include the need for a relatively large lesion within a voxel, the lack of technical standardization in terms of acquisition techniques, and volume averaging (Burtscher et al., 2000; Villanueva-Meyer et al., 2017). Furthermore, low concentration of certain metabolites relative to water molecules makes detection of certain substances at clinical fields <3 T difficult, and can also lead to long acquisition times (Wu et al., 2016).

One novel MR technique that can also be used to investigate tissue metabolites is that of chemical exchange saturation transfer (CEST). CEST is capable of detecting a chemical compound of interest based on the exchange of magnetization between liable hydrogen protons of said compound and surrounding water molecules. This property allows for the detection of certain tissue metabolites with a higher spatial resolution than MRS (Hoefemann et al., 2021). In the case of glioma imaging, amide proton transfer (APT) CEST is the most common application of CEST, although not as widely available or commonly used as MRS. Elevated concentrations of proteins in glioma compared to surrounding tissues and the high rates of intracellular proton exchanges leads to an increased APT level. APT CEST can be used to differentiate between LGGs and HGGs (Choi et al., 2017), differentiation between regions of tumor and peritumoral edema (Wen et al., 2010), and is gaining interest as a method of investigating intratumoral heterogeneity (Warnert et al., 2021). One notable advantage of MRS over CEST is that MRS is able to simultaneously quantify multiple compounds, while CEST is only able to acquire one or two compounds at a time. Overlapping CEST effects from multiple metabolites also causes low specificity in CEST imaging for the measurement of specific tissue metabolites. The combination of CEST and MRS is currently being investigated, and may play a role in glioma imaging (Hoefemann et al., 2021).

### Positron Emission Tomography as a Tool for Preoperative Planning

Positron emission tomography (PET) uses a variety of radio-labeled tracers to assess rates of cell proliferation, glucose metabolism, amino acid uptake, and membrane biosynthesis. This emerging clinical tool in the field of neuro-oncology thus provides valuable molecular, functional, and metabolic information about tumor biology. The most common molecular tracers utilized for PET imaging of gliomas include those that image glucose metabolism—2-^18^F-fluoro-2-deoxy-D-glucose (^18^F-FDG)—and those that image amino acid transport— O-(2-^18^F-fluoroethyl)-L-tyrosine (^18^F-FET), (S-^11^C-methyl)-L-methionine (^11^C-MET), and 3,4-dihydroxy-6-^18^F-fluoro-L-phenylalanine (^18^F-FDOPA) (Albert et al., 2016).

Positron emission tomography imaging with the glucose analog ^18^F-FDG compares rates of cellular ^18^F-FDG uptake in tumor cells relative to surrounding normal brain parenchyma. A high degree of ^18^F-FDG PET uptake correlates with higher tumor grade and decreased survival (Padma et al., 2003). In the stage of primary tumor diagnosis, ^18^F-FDG PET can also be used to distinguish between GBM and CNS lymphomas given markedly distinct rates of ^18^F-FDG uptake between the two malignancies (Kosaka et al., 2008; Yamashita et al., 2013; Fink et al., 2015). However, there are significant limitations to the use of ^18^F-FDG PET for glioma diagnosis. The high rate of glucose metabolism and ^18^F-FDG uptake in normal brain cortex limits the diagnostic accuracy of ^18^F-FDG PET (Albert et al., 2016). Moreover, this modality has limited specificity in distinguishing between gliomas, metastatic lesions, and even other non-neoplastic lesions like brain abscesses, neurosarcoidosis, and certain demyelinating CNS disorders (Omuro et al., 2006; Albert et al., 2016). Therefore, ^18^F-FDG is not typically used in the presurgical planning period, instead amino acid PET tracers (AA PET) are preferred molecular tracers.

Tumor cells take up AA PET tracers at a higher rate than surrounding brain parenchyma; therefore, these tracers can provide greater tumor-to-background contrast than ^18^F-FDG (Albert et al., 2016). Histology-validated series show that ^18^F-FET, ^11^C-MET, and ^18^F-FDOPA are superior tracers to ^18^F-FDG PET in delineating extent of glioma infiltration in both LGGs and HGGs. Of note, ^18^F-FET is commonly used in the context of glioma characterization given that it has a high sensitivity in detection of WHO grade III and IV gliomas with the majority (>95%) of these tumors displaying high tracer uptake (Hutterer et al., 2013; Rapp et al., 2013; Jansen et al., 2015; Albert et al., 2016).

AA PET is also effective in visualizing tumor volumes that extend past areas of contrast enhancement on T1, and can also delineate tumor infiltration within non-specific areas of abnormal T2/FLAIR enhancement (Chen et al., 2006; Galldiks et al., 2006, 2011; Ledezma et al., 2009; Pafundi et al., 2013; Dunet et al., 2016). Furthermore, studies focused on examining intratumoral heterogeneity also show that AA PET is capable of identifying areas of higher cell proliferation within heterogenous glioma (Tanaka et al., 2009; Ewelt et al., 2011). AA PET thus could be used in the clinical setting to help optimize image-guided biopsy in the preoperative period by identifying areas of high cellularity and proliferation, and could also serve a role maximizing EOR by identifying areas of infiltrating glioma not seen on anatomic MRI sequences (Ledezma et al., 2009).

**FIGURE 7:**
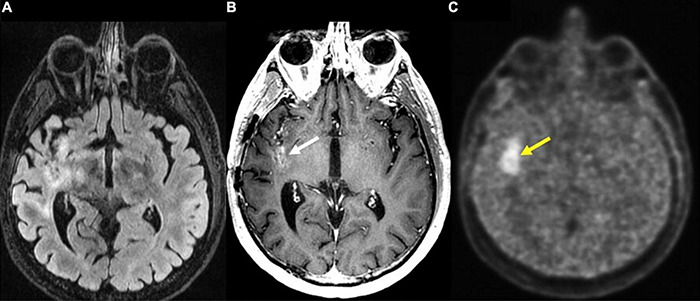
Recurrent glioblastoma in right posterior insula (FMISO PET-MR). **(A)** Axial FLAIR: Ill-defined hyperintense area in the right posterior insula (black arrow); **(B)** axial T1 post-contrast: mild enhancement in the right posterior insula (white arrow); **(C)** axial ^18^F-FMISO PET: avid uptake of FMISO tracer in the right posterior insular (yellow arrow). Biopsy targeted to this region showed recurrent glioblastoma. ^18^F-FMISO, Fluoromisonidazole; PET, Positron emission tomography.

PET can also be used as a tool for prognostication. Despite previously stated limitations of ^18^F-FDG PET, this modality is reported to correlate with increased survival in patients with new GBM diagnosis (Albert et al., 2016; Sun et al., 2018). In the case of AA PET scans, earlier decrease in time-activity curves in dynamic (kinetic) ^18^F-FET PET correlates with malignant transformation in the case of WHO grade II gliomas, and decreased overall survival in astrocytic HGGs (Jansen et al., 2015). Additionally, ^18^F-fluoromisonidazole (^18^F-FMISO), a PET imaging agent that selectively binds to hypoxic tissues, that is used to visualize degree of GBM tissue hypoxia also holds potential for prognostication (Figure 7). Given that higher pre-treatment ^18^F-FMISO standardized uptake value peak (SUV_peak_) is significantly associated with shorter OS (Gerstner et al., 2016).

Overall, PET is a powerful tool that has great clinical potential that is currently not commonly used in the preoperative planning period due to a lack of widespread utilization of AA PET in imaging centers, cost of use, and differences in methodology among studies investigating clinical use of PET (Albert et al., 2016).

## Adjuncts to Assist With Intraoperative Decision Making

Surgical planning through the use of the aforementioned MR modalities is essential for tumor characterization and localization. Gliomas can often involve brain structures that are critical for normal sensorimotor, visual, and cognitive function. Therefore, there must be a balance between resecting as much tumor as possible and preserving regions that are critical to optimize patient quality of life. In the following section, we describe MR and non-MR techniques that can help identify areas that are critical for neuronal function and thus guide preoperative and intraoperative decision making to maximize the safe resection of gliomas.

### Diffusion Tensor Imaging

Diffusion tensor imaging (DTI) is a variant of DWI that also measures Brownian motion of water molecules but does so along a greater number of orthogonal planes. DTI can measure the directionality in diffusion of water molecules, a property that is particularly useful for the detection of large white matter tracts. The corticospinal tract, arcuate fasciculus, optic radiations, and fronto-occipital fasciculus are composed heavily myelinated axons that travel in parallel bundles, an architectural property that promotes the diffusion of water molecules along the direction of these white matter tracts (Cho and Jang, 2020). Furthermore, DTI calculates the metric of fractional anisotropy (FA), a numerical value based on the anisotropy of water molecules along axons that can provide insight into presence of intact myelinated white matter tracts (Le Bihan et al., 2001; Oh et al., 2005). For example, substantial decreases in FA are known to correlate with disruptions in white matter due to the presence of gliomas and peritumoral edema (Lu et al., 2004; Zolal et al., 2012; Fudaba et al., 2014).

**FIGURE 8:**
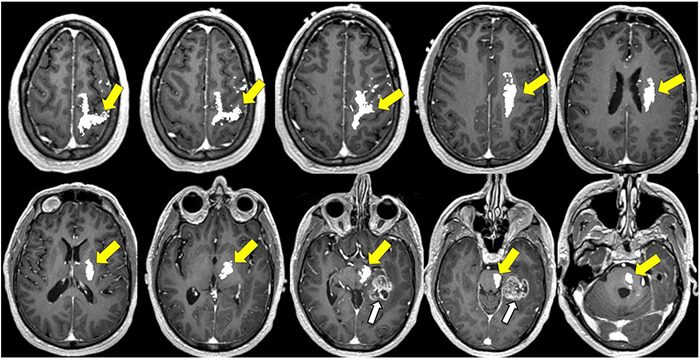
Tractography corticospinal tract. Corticospinal tractography (yellow arrows) spanning from the superior motor cortex to pons overlaid on axial T1 post-contrast images showing enhancing necrotic glioblastoma (white arrows) in the left posterior parahippocampal gyrus.

**FIGURE 9:**
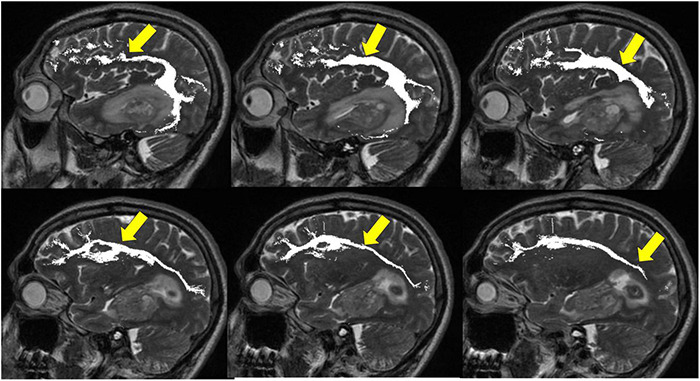
Tractography: arcuate fasciculus. Arcuate fasciculus tractography (yellow arrows) overlaid on sagittal T2 images.

**FIGURE 10:**
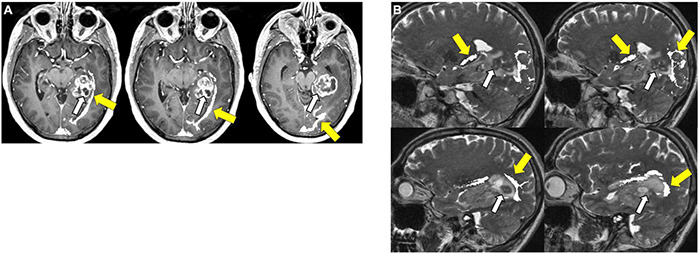
Tractography: optic radiations. **(A)** Optic radiation tractography (yellow arrows) overlaid on axial T1 post-contrast images showing enhancing necrotic glioblastoma (white arrows) centered in the left posterior parahippocampal gyrus; **(B)** optic radiation tractography (yellow arrows) overlaid on sagittal T2 images.

**FIGURE 11:**
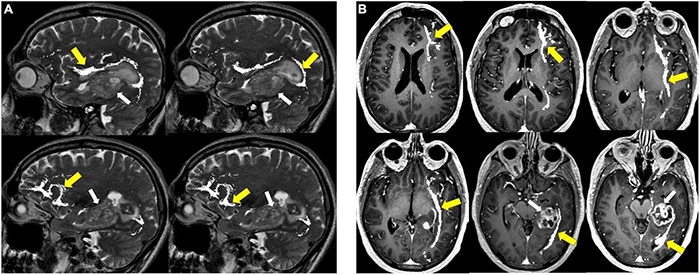
Tractography: inferior fronto-occipital fasciculus. **(A)** Inferior fronto-occipital fasciculus tractography (yellow arrows) overlaid on sagittal T2 images. Left temporal glioblastoma (white arrows) is adjacent to but does not invade the tract. **(B)** Inferior fronto-occipital fasciculus tractography (yellow arrows) overlaid on axial T1 post-contrast images. Left temporal glioblastoma (white arrows) is adjacent to but does not invade the tract.

**FIGURE 12:**
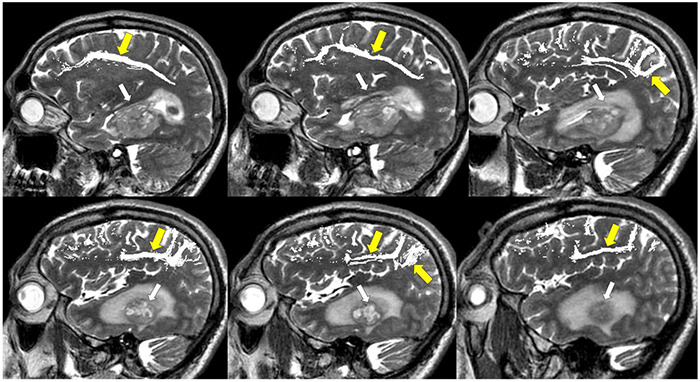
Tractography: superior longitudinal fasciculus. Sagittal T2-weighted images show glioblastoma centered in the hippocampus and parahippocampal gyrus (white arrows). Overlay of tractography of superior longitudinal fasciculus (yellow arrows) demonstrates sparing of the tract by the tumor.

The ability of DTI to delineate differences between subcortical white matter is useful during intra-operative navigation for identifying white matter fiber tracts and for defining the proximity to these anatomical tracts at the tumor resection margins (Figures 8–12; Berman et al., 2007; Zolal et al., 2012). One randomized controlled trial (RCT) comparing outcomes with and without preoperative DTI in 214 diffuse glioma patients with pyramidal tract involvement demonstrated that the use of DTI-based neuronavigation intraoperatively can help maximize safe rates of gross total resection (GTR) and improve outcomes for patients with low- and high-grade gliomas involving the pyramidal tract with significant post-operative functional benefits (Wu et al., 2007; Verburg and de Witt Hamer, 2021).

There are several limitations to this imaging modality when using it intra-operatively to assist with tumor resections. First, there can be great variability of tracking algorithm settings that can lead to white matter tract overestimation. Second, this modality can be user-dependent depending on region-of-interest placement (Verburg and de Witt Hamer, 2021).

One additional significant limitation of the use of DTI (and of any imaging modality where a preoperatively generated image is used for intraoperative neuronavigation) lies in the phenomenon of “brain shift” where the brain shifts intraoperatively away from the dural edge, and the spatial relationship of the brain is altered compared to the pre-operative images (Kuhnt et al., 2012). Glioma tumor volume has a significant correlation with brain shift, with one study reporting shift relative to preoperative imaging as high as 14.3 mm (Reinges et al., 2004). There is further evidence to suggest that length of surgery, craniotomy size, and supratentorial location correlate with greater brain shift (Reinges et al., 2004; Reinertsen et al., 2014). Intraoperative MRI (iMRI) has can be employed as a supplement to DTI and functional imaging as a method of compensating for brain shift and assessing the degree of tumor resection. Multiple studies show that this method increases EOR in glioma surgery with no significant increase in new postoperative deficits (Senft et al., 2011; Kuhnt et al., 2012).

### Functional MRI

Functional MRI (fMRI) can be used to visualize neuronal activity by measuring the ratio of deoxyhemoglobin to oxyhemoglobin to generate a blood oxygen level dependent (BOLD) signal that allows for the spatiotemporal mapping of neuronal activity during periods of rest or cognitive tasks (Buchbinder, 2016). BOLD signal is determined by changes in the magnetic field surrounding red blood cells that depends on the state of oxygenation of hemoglobin. Oxyhemoglobin is diamagnetic and has a similar magnetic field relative to surrounding brain tissue. On the other hand, deoxyhemoglobin- which is at a higher concentration in brain tissue of high neuronal activity- is paramagnetic and forms local endogenous magnetic field gradients with strength that is dependent on deoxyhemoglobin concentration. These magnetic field gradients can be detected on T2 and T2* sequences (Thulborn et al., 1982; Ogawa et al., 1993). fMRI can be used in presurgical functional brain mapping, particularly when localizing areas of motor function (Krings et al., 2002; Bush et al., 2017).

Despite its utility in preoperative planning, a variety of challenges arise with the use of fMRI for glioma resection. First, vascular changes in HGGs can lead to neurovascular decoupling and BOLD signal loss that may not actually be reflective of absence of neuronal function, thus resulting in false negative fMRI signal loss (Fujiwara et al., 2004). Given this vulnerability of fMRI signal to microstructural alterations, task-based fMRI is reported to be more reliable in LGGs than HGGs (Castellano et al., 2017). Another limitation is that, compared to direct electrical stimulation (DES), fMRI is significantly limited in mapping the functional connectivity of language areas with sensitivity and specificity of 91 and 64% for identification of Broca’s area and 93 and 18% for identification of Wernicke’s area (Hervey-Jumper and Berger, 2016). Moreover, there is considerable variability between studies in the reported sensitivity and specificity for language mapping, a factor that can be attributed to differences in language paradigms investigated, a lack of standardization of distance thresholds when utilizing DES to confirm fMRI accuracy, and glioma grade heterogeneity (Morrison et al., 2016; Ellis et al., 2020).

Another important factor to consider when utilizing fMRI for clinical decision making is that sub-regions of functional areas are activated depending on a specific task, and while fMRI mapping suggests that certain areas are involved in particular tasks, it does not indicate whether said areas are necessary for function (Haller and Bartsch, 2009). Thus, surgical sparing of certain functional areas based on fMRI can often times preclude what may be a safe resection (Southwell et al., 2018). Therefore, while fMRI can be an adjunct for assessing surgical risk of tumor resection preoperatively, DES remains the gold standard for determining the location of function intraoperatively (Haller and Bartsch, 2009; Borchers et al., 2011; De Witt Hamer et al., 2012; Desmurget and Sirigu, 2015; Weiss Lucas et al., 2020).

### Magnetoencephalography

Magnetoencephalography (MEG) detects magnetic fields that are generated by electrical currents from neuronal action potentials. MEG can be registered with a 3D MRI sequence to visualize functional neuronal activity with high spatiotemporal resolution- a spatial resolution of a few millimeters and a temporal resolution in milliseconds (Naeije et al., 2016). In addition to providing information regarding task-based neuronal activity, MEG is able to identify regions of high functional connectivity (HFC) and low functional connectivity (LFC) (Verburg and de Witt Hamer, 2021). This serves as a useful method of preoperatively evaluating function of tumoral and peritumoral brain tissue for surgical planning, particularly for preoperative somatosensory and motor mapping for which there are multiple validation studies (Yang et al., 1993; Schiffbauer et al., 2003; Lin et al., 2006; Nagarajan et al., 2008; Lee et al., 2020). Furthermore, MEG connectivity maps can reliably identify areas lacking in eloquent cortex, for LFC there is reportedly a negative predictive value of 100% for the presence of eloquent cortex during intraoperative DES (Martino et al., 2011). Therefore, MEG can detect areas near functional cortex that are amenable to resection while minimizing neurological deficits (Guggisberg et al., 2008).

Additionally, there is evidence to suggest that MEG may serve as an important supplement to DES during tumor resection in areas involving language processing. One study using MEG mapping to aid in LGG and HGG tumor resection found that surgical resection of HFC sites with negative response to intraoperative DES correlated with early transient postoperative functional decline in language processing that resolved within 3 months in patients who did not experience additional neurological insult such as stroke or early tumor progression. Although limited by a small sample size, these findings suggest that MEG can serve as a predictor of early transient decline in language processing following glioma resection (Lee et al., 2020). This modality is limited by cost, given that liquid helium is required to maintain the superconducting equipment of MEG machines, and availability is considerably limited (Hervey-Jumper and Berger, 2016).

The reliability of MEG in relation to fMRI as a method of functional mapping is an active area of research. One ongoing clinical trial reports that MEG has a higher specificity for motor and language mapping but a lower sensitivity for motor mapping than fMRI. Furthermore, this study reports that using MEG and fMRI in combination could potentially serve to increase the accuracy of motor mapping relative to the use of MEG and fMRI separately. While published data for this study has a low sample size, ongoing data collection may provide further insights into the clinical utility of combining MEG and fMRI (Ellis et al., 2020).

### Navigated Transcranial Magnetic Simulation

Navigated transcranial magnetic stimulation (nTMS) is a technique that utilizes a wound copper coil to generate strong magnetic pulses targeting a specific area of the brain. This coil is paired with a stereotactic image-guided system to generate highly accurate functional maps capable of differentiating eloquent from non-eloquent tissue through non-invasive stimulation and inhibition of specific cortical areas (Bush et al., 2017). nTMS is well-suited for preoperative identification of eloquent motor cortex, with a reported accuracy of nTMS for generating a functional motor map in relation to the gold standard, DES, of 99.7% (Picht, 2014). Importantly, the high accuracy of nTMS for generating a functional motor map is reported to be consistent between different examiners in the clinical setting (Picht et al., 2009; Forster et al., 2011; Krieg et al., 2012; Paiva et al., 2012; Tarapore et al., 2012; Picht, 2014; Haddad et al., 2020). In practice, nTMS influences preoperative plans for tumor resection by confirming or negating suspected involvement of primary motor cortex by the tumor, often leading to improved surgical outcomes for tumors involved with motor pathways (Frey et al., 2014; Picht, 2014; Picht et al., 2016). Furthermore, nTMS can be utilized to enhance accuracy of white matter tractography when used in conjunction with DTI. This technique can help diminish intraoperative injury in patients whose white matter tracts are closely involved with tumor, especially there are significant signal alterations in DTI due to peritumoral edema and vascular changes (Weiss et al., 2015). There is an abundance of clinical evidence to support favorable clinical outcome in patients receiving preoperative nTMS for resection of lesions involving motor cortex including greater improvement in postoperative motor function, lower rates of postoperative motor decline, and increased GTR (Krieg et al., 2014; Raffa et al., 2019). Therefore, employing nTMS for surgical planning in cases where gliomas involve motor cortex is an important consideration.

Language mapping is more challenging with this technology and usually relies on navigated repetitive TMS (nrTMS), which utilizes repetitive bursts of TMS and is better-suited for functional language mapping than the single pulses utilized for nTMS (Haddad et al., 2020). Similar to other functional mapping techniques, nrTMS has great variability in reported sensitivity and specificity for language mapping between studies (Picht et al., 2013; Tarapore et al., 2013). There are studies to suggest that combination of functional modalities such as fMRI or DTI with nrTMS may lead to improved functional mapping and clinical outcomes, but further investigation is needed to make these practices commonplace in the clinical setting (Ille et al., 2015; Könönen et al., 2015; Sollmann et al., 2018).

## Imaging for Monitoring Treatment Response and Tumor Recurrence

Despite the aggressive standard of care, nearly all diffuse gliomas eventually recur and postoperative surveillance imaging is critical to identify tumor recurrence as early as possible to provide treatment that can help slow down the rate of disease progression (Stupp et al., 2005, 2014; Le Rhun et al., 2016). There are several studies that show high rates of recurrence among LGGs within the 5 year postoperative period despite gross total tumor resection (Soffietti et al., 1998; Shaw et al., 2008; Chaichana et al., 2010). Early diagnosis of tumor recurrence in LGGs is critical to diminish chances of malignant transformation of recurrent LGGs (Duffau and Taillandier, 2015; Le Rhun et al., 2016). In the case of GBM, tumor recurrence is inevitable and can be difficult to manage. Common practice to attempt to manage recurrent GBM is the use of antiangiogenic agents (i.e., bevacizumab), nitrosourea alkylating agents, and/or repeat tumor resection (Wick et al., 2010; Batchelor et al., 2013; Johnson et al., 2013).

**FIGURE 13:**
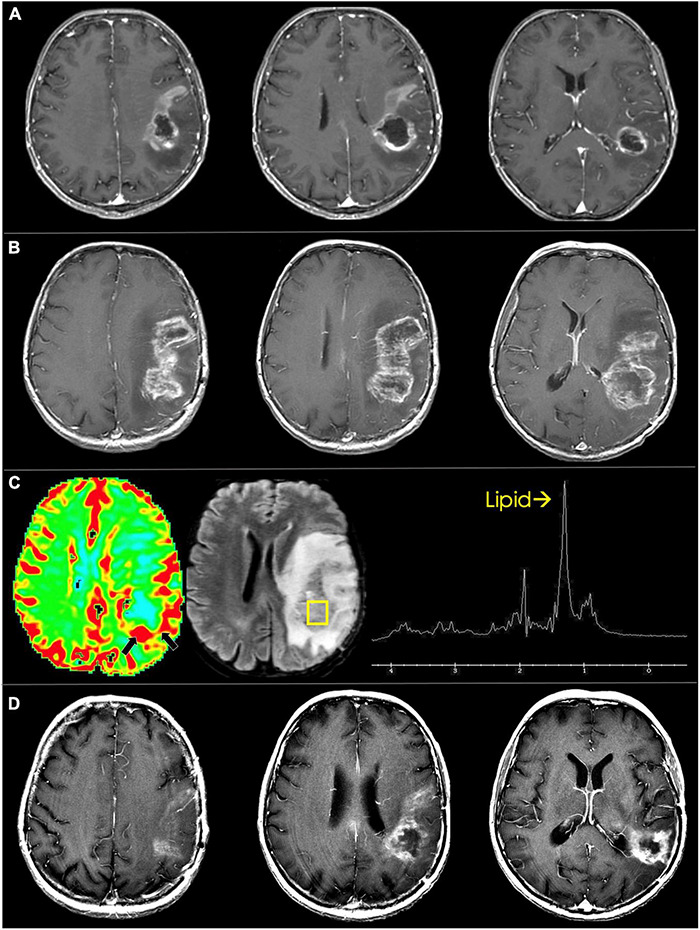
Pseudoprogression in glioblastoma. **(A)** Immediate pre-radiotherapy: axial T1 post-contrast images show rim enhancing and centrally necrotic left frontoparietal glioblastoma. **(B)** Eight-week follow up: Immediate post-radiotherapy axial T1 post-contrast images show marked increase in enhancement and necrosis. **(C)** Dynamic susceptibility-weighted contrast-enhanced perfusion MRI shows mild increase in blood volume along the posterior rim (black arrows). Single voxel proton spectroscopy targeted to the posterior component shows markedly increased lipid peak suggesting tissue necrosis. **(D)** Three-months follow up: axial T1 post-contrast images show marked decrease in enhancement and necrosis of the treated glioblastoma.

Postoperative surveillance for tumor recurrence and treatment-related changes is an ongoing challenge given that imaging characteristics of glioma recurrence and gliosis or treatment effect are similar in appearance on T1 and T2 sequences (Albert et al., 2016). Hyperintense signal on T1 post-contrast imaging within the resection cavity in the postoperative period is concerning for tumor recurrence, but can also be representative of ischemic brain tissue as well as devitalized tumor (Winter et al., 2020). Furthermore, inflammatory processes following chemoradiation can lead to a transient edematous process that can mimic signs of tumor recurrence in both HGG and LGG patients for several months after therapy. This process is often mistaken for tumor progression but does not represent true tumor recurrence and is thus termed pseudoprogression (Figure 13; Winter et al., 2020; Li et al., 2021).

### Response Assessment in Neuro-Oncology Criteria for Differentiating Tumor Recurrence From Pseudoprogression

In an effort to distinguish tumor progression and pseudoprogression on imaging, the updated Response Assessment in Neuro-Oncology (RANO) criteria were developed in 2010. The RANO criteria state that tumor progression taking place during the 12 week period following the completion of radiotherapy can only be identified through imaging if new tumor enhancement arises outside of the radiation field (Wen et al., 2021). Clues to true progression cited in other articles include increased T2/FLAIR signal extending past the radiation field as well as involvement of the corpus callosum, signal that crosses midline, and subependymal involvement (Mullins et al., 2005; Abel et al., 2012; Strauss et al., 2019). Serial imaging over time can attempt to distinguish pseudoprogression from true tumor recurrence. Pseudoprogression on serial imaging is characterized by loss of post-contrast enhancement signal or volume over time (Li J. et al., 2020). However, this can be flawed as post-treatment effects slowly enlarge, usually over the span of the first 3–6 months after postoperative radiation therapy, and require many serial scans before finally regressing (Figure 13). The fact that the definitive distinction between pseudoprogression and tumor progression usually takes months, there is a risk that patients are under- or over-treated (Ellingson et al., 2017b). Obtaining early post-operative MRI scans (i.e., within 48 h of surgery) is critical for differentiating between pseudoprogression and true progression to avoid postsurgical confounders, such as tissue ischemia, that may alter signal intensity or enhancement on subsequent MRI scans. However, in most studies the sensitivity and specificity of anatomic (T1 and T2 weighted) MR sequences for detection of HGG progression are only 68 and 77%, respectively. Therefore, additional imaging modalities, like MR perfusion imaging, DWI and MRS, can be employed to aid in the diagnosis (van Dijken et al., 2017).

### Advanced MR Sequences for Monitoring Tumor Recurrence

MR perfusion imaging is widely used to aid in distinguishing between recurrence and treatment effect as recurrent HGG exhibits higher rCBV values relative to post-treatment radiation effects (Sadeghi et al., 2008; Schmainda, 2012; Choi et al., 2013; Patel P. et al., 2017). DWI also plays a role in differentiating recurrent glioma from radiation treatment effects given that cell density is high in recurrent glioma tumors and low in areas of treatment changes. This translates to smaller ADC values in true glioma recurrence groups relative to pseudoprogression and post-radiation changes groups (Li C. et al., 2020). MRS can also be used to differentiate glioma from treatment effects. Particularly for HGGs, MRS shows an elevated Cho/Cr ratio in areas of tumor recurrence compared to areas of radiation-induced necrosis or treatment effect. There are also reported differences in Cho/NAA ratios between recurrent tumor and areas of radiation-induced necrosis (Chuang et al., 2016). Despite these imaging modalities being more effective than anatomic MRI in helping distinguish between pseudoprogression, treatment effect, and true progression, their accuracy and efficacy is limited and often surgical biopsy with histopathological analysis is needed to confirm the diagnosis (Li et al., 2021).

### Positron Emission Tomography Imaging for Monitoring Tumor Recurrence

Positron emission tomography also plays a role in the postoperative surveillance. Given that there is evidence to suggest that reduction in amino acid uptake is indicative of treatment response (Sun et al., 2018), there is interest in the use of AA PET as a method of facilitating the distinction between glioma recurrence and pseudoprogression (Galldiks et al., 2006; Jansen et al., 2013). Furthermore, ^18^F-FET PET can be used to identify pseudoprogression within the first 3 months following chemoradiation therapy with an accuracy of 96% (Galldiks et al., 2015). Regarding the distinction between glioma recurrence and treatment-related changes there is evidence to suggest that the diagnostic accuracy of 11C-MET PET and ^18^F-FET PET for differentiating recurrence from treatment-related changes is high with a sensitivity and specificity of 91 and 100% for both tracers (Grosu et al., 2011; Cui et al., 2021). Furthermore, a lower tumor-to-normal-uptake (T/N) ratio during AA PET analysis is associated with lower recurrence and longer OS (Galldiks et al., 2015; Patel P. et al., 2017; Deuschl et al., 2018). The use of ^18^F-FDG for detection of tumor recurrence remains controversial, similar to its use at the time of diagnosis, but its wide availability warrants further investigation to attempt to optimize its clinical use (Wang et al., 2015).

## Imaging to Assess for Pseudoresponse Following Immunotherapy

Pseudoresponse refers to a decrease in contrast extravasation due to diminished leakiness of the BBB, resulting in markedly diminished CE on T1 post-contrast imaging. This is a finding that develops following treatment with antiangiogenic agents such as bevacizumab. Patients with GBM recurrence receiving bevacizumab immunotherapy. Anti-VEGF-A properties of bevacizumab often lead to a substantial, rapid radiologic response of contrast enhancement reduction and decreased edema just days after treatment; however, current evidence indicates that bevacizumab does not confer a benefit in OS (Norden et al., 2008). This is likely due to tumor adaptation to antiangiogenic therapy resulting in a hypoxic NCE invasive tumor phenotype that is capable of surviving despite decreased vascular proliferation. A return to CE from this period of NCE is associated with particularly poor outcome (Iwamoto et al., 2009; Galanis et al., 2012; Kim et al., 2015; Wick et al., 2016); therefore, an active area of investigation is geared toward developing a generalizable protocol for the identification of pseudoresponse in GBM.

### Response Assessment in Neuro-Oncology Criteria for Assessing Pseudoresponse

The updated RANO criteria recommend the use of T2/FLAIR to assess for pseudoresponse, defined by the RANO criteria as an area of >50% reduction in CE without a significant decrease in the presence of NCE on T2/FLAIR. The RANO criteria provide an essential framework for the standardization of glioma surveillance following antiangiogenic therapy. Despite the existence of studies suggesting that DWI, MRS, and PET scans may play a valuable role in the identification of pseudoprogression, general experience with the use of these modalities for this purpose remains limited, and standardization of postoperative surveillance through incorporation of these modalities to the RANO criteria is an ongoing challenge (Boxerman et al., 2018).

One area of active investigation that shows how findings from the RANO group are being expanded upon is shown through immunotherapy response assessment in neuro-oncology (iRANO) criteria. iRANO integrates the framework of response assessment that the RANO group established for workup of LGG and HGG to establish guidelines for interpreting initial progressive imaging findings in patients with LGGs and HGGs on immunotherapy with goals of optimizing immunotherapy regimens (Okada et al., 2015). There may be a role in the incorporation of iRANO criteria in the context of pseudoresponse as these criteria continue to be refined and updated.

### Positron Emission Tomography as a Tool for Assessing Pseudoresponse

Positron emission tomography also plays a role in differentiating pseudoresponse from treatment response in patients with GBM recurrence receiving bevacizumab immunotherapy. Anti-VEGF-A properties of bevacizumab often lead to a substantial, rapid radiologic response of contrast enhancement reduction and decreased edema just days after treatment; however, current evidence indicates that bevacizumab does not confer a benefit in OS (Norden et al., 2008). A large fraction of patients that originally exhibit radiological response eventually develop disease progression that can be tracked by increases in T2/FLAIR signal hyperintensities; however, objective measures of T2 FLAIR changes are challenging employ reliably (Boxerman et al., 2018). AA PET has been reported as a potentially viable adjunct in analyzing pseudoresponse. As a prognostication tool in the context of pseudoresponse, AA PET shows promise with existing evidence showing that persistent ^18^FET-PET signal on NCE tumor during bevacizumab treatment is predictive of a significant decrease in OS (Wirsching et al., 2021).

## Emergence of Radiomics

Radiomics is the practice of extracting and analyzing quantitative information from diagnostic images in a manner that can track subtleties in tumor characteristics and complex patterns that are difficult to recognize by the human eye to produce prognostically important information such as treatment response vs. the likelihood of tumor progression and survival estimates (Mayerhoefer et al., 2020). Additionally, radiomics may be a method of characterizing heterogeneity throughout the entire tumor volume, compared to tumor biopsies that only capture heterogeneity within a specific, local region (Mayerhoefer et al., 2020). While detailing all of the different radiomics methodologies that can be employed to study tumor composition is beyond the scope of this review (Hu et al., 2020; Mayerhoefer et al., 2020), there are existing radiomics methods that have been employed to study intratumoral characteristics in the context of GBM. One example lies in one study where image-guided regional biopsies of GBM were collected, regional GBM driver genes were identified, and biopsy sites were co-registered with MRI and texture maps to match genetic regional status with specific imaging measurements (Hu et al., 2017). Machine learning algorithms were then employed to identify MRI signatures at the voxel level that correlated with GBM driver gene status within different regions of GBM tumor (Hu et al., 2017). This type of technique can thus serve to produce an imaging algorithm that can potentially capture tumoral molecular markers based on stereotyped imaging patterns.

### Radiomics and Recurrence

One example of an integrated radiomics model for discriminating tumor recurrence from radiation necrosis in glioma patients has recently been developed by Wang et al. (2020). This model was developed in a primary cohort of 112 patients with pathologically confirmed gliomas and was validated by a cohort of 48 additional glioma patients. ^18^F-FET PET and ^11^C-MET PET along with individualized patient data and characteristics were utilized to generate a model predicting tumor recurrence. The integrated model consisted of 15 features that were identified as significant predictors of recurrence (*p* < 0.001) including variables like mean tumor-background ratio (TBR) of ^18^F-FET, maximum TBR of ^11^C-MET and patient age as well as a radiomics signature, was found to be highly predictive of recurrent glioma and accurate across both test and validation cohorts (Wang et al., 2020). More work will be needed to confirm radiomics based models are generalizable across different institutions with different scaling parameters, but this is an exciting emerging field that is advancing understanding of glioma behavior and developing valuable prognostic information in a non-invasive manner. Although there are still significant strides that remain to be made before broad clinical applications of emerging radiomics models, these are important initial steps in advancing individualized patient care for management of gliomas.

## Conclusion

MR and PET scans have revolutionized the management of glioma patients. Upon diagnosis of intra-axial brain tumor, it is standard of care to obtain T1, T2/FLAIR, DWI, and susceptibility images to guide preoperative planning. Increasingly, this practice is being enhanced further through the use of advanced imaging modalities such as MR perfusion, diffusion, and spectroscopy and PET scans. Moreover, surgical resections are assisted by functional imaging assessments and intra-operative use of anatomical images and neuronavigation to maximize the safety of glioma resection. The monitoring of tumors during treatment through serial imaging is of great importance, but can be challenging, as distinguishing between treatment response and progression is often difficult. In the future it will be important to standardize the use of sequences like MR perfusion, diffusion, and spectroscopy and PET scans for postoperative tumor surveillance as they can dramatically improve interpretation of the underlying biologic process. Lastly, radiomics is emerging as an exciting big data tool for quantifying the information provided in the images and thereby potentially improving precision and accuracy, however the generalizability and role for radiomics for the management of gliomas is still unanswered.

## Author Contributions

All authors contributed to the manuscript and approved the final version of the manuscript.

## Conflict of Interest

The authors declare that the research was conducted in the absence of any commercial or financial relationships that could be construed as a potential conflict of interest.

## Publisher’s Note

All claims expressed in this article are solely those of the authors and do not necessarily represent those of their affiliated organizations, or those of the publisher, the editors and the reviewers. Any product that may be evaluated in this article, or claim that may be made by its manufacturer, is not guaranteed or endorsed by the publisher.

## Abbreviations

EOR: extend of resection
HGG: high-grade glioma
LGG: low-grade glioma
GBM: glioblastoma
MRI: magnetic resonance imaging
FLAIR: fluid-attenuated inversion recovery
BBB: blood-brain barrier
CE: contrast-enhancing
NCE: non-contrast enhancing
IDH: isocitrate dehydrogenase
MGMT: O6-methylguanine-DNA methyltransferase
DWI: diffusion-weighted imaging
ADC: apparent diffusion coefficient
SWI: susceptibility-weighted imaging
DSC: dynamic susceptibility contrast
DCE: dynamic contrast enhanced
ASL: arterial spin labeling
rCBV: relative cerebral blood volume
MRS: MR spectroscopy
DTI: diffusion tensor imaging
FA: fractional anisotropy
RCT: randomized controlled trial
GTR: gross total resection
iMRI: intraoperative MRI
fMRI: functional MRI
BOLD: blood oxygen level dependent
DES: direct electrical stimulation
MEG: magnetoencephalography
HFC: high functional connectivity
LFC: low functional connectivity
nTMS: navigated transcranial magnetic stimulation
nrTMS: navigated repetitive TMS
PFS: progression-free survival
OS: overall survival
PET: positron emission tomography
^18^F-FDG: 2-^18^F-fluoro-2-deoxy-D-glucose
^18^F-FET: O-(2-^18^F-fluoroethyl)-L-tyrosine
^11^C-MET: (S-^11^C-methyl)-L-methionine
^18^F-FDOPA: 3,4-dihydroxy-6-^18^F-fluoro-L-phenylalanine
AA PET: amino acid PET
TBR: tumor-background ratio.
